# Perioperative management of pulmonary arterial hypertension in children undergoing congenital heart surgery: a systematic review and meta-analysis

**DOI:** 10.1186/s13019-026-03893-5

**Published:** 2026-04-25

**Authors:** Eberechukwu Ikwuanusi, Tanisha Rajah, Katie Scandrett, Evans Atiah Asamane, Sara Volpi

**Affiliations:** 1 University Hospitals Birmingham, Birmingham, UK; 2 Birmingham Medical School, University of Birmingham, Birmingham, B15 2TT UK; 3 University of Birmingham, Birmingham, UK; 4 School of Medicine, Keele University, Keele, Newcastle, ST5 5BG UK; 5 Department of Cardiac Surgery, St. Thomas’ Hospital London, London, UK

**Keywords:** Congenital heart disease, Pulmonary arterial hypertension, Systematic review, Meta-analysis

## Abstract

**Background:**

Children with pre-existing pulmonary arterial hypertension (PAH) undergoing surgery for congenital heart disease (CHD) are at an increased risk of morbidity and mortality, primarily due to complications associated with increased pulmonary arterial pressures. Despite the clinical significance of this risk, no comprehensive review of perioperative strategies to manage pre-existing PAH in paediatric CHD patients undergoing cardiac surgery exists. This systematic review aims to address this gap.

**Methods:**

A comprehensive systematic literature search was conducted on Scopus, Medline, Embase, PubMed, Cochrane Library and grey literature; studies were screened using eligibility criteria. Data was extracted using a pre-tested standard form and the methodological quality appraised using the RoB 2 tool. A meta-analysis was used to analyse, summarise, and interpret the extracted data.

**Results:**

Ten randomised controlled studies were included in this review, comprising 520 patients, ages ranged from 0.21 to 13.8 years across 6 countries. All studies reported a greater decrease in postoperative mean pulmonary arterial pressure (mPAP) in the intervention groups compared to the control groups. Although perioperative management did not significantly reduce postoperative PAP (pooled mean difference, -0.44; 95% CI: -4.62-3.73; I^2^ = 88.73%), their use resulted in statistically significant decreases in mean ICU stays (pooled mean difference, -1.08; 95% CI: -1.90-0.25, p-value = 0.01; I^2^ = 86.21%) and ventilation times (pooled mean difference, -13.29; 95% CI: -25.78 -0.80, p - value = 0.04; I^2^ = 97.47%) compared to controls. Sildenafil was the most used intervention, with a significantly greater reduction in PAP compared to other management strategies (pooled mean difference, -6.27; 95% CI: -8.982- -3.57, p-value < 0.001; I^2^ = 49.84%). Among studies reporting pulmonary hypertensive crises and mortality, the prevalence of pulmonary hypertensive crises was 6.18% in the control group versus 3.43% in the treatment group. Mortality rates were 1.27% in the control group and 0.54% in the treatment group, respectively.

**Conclusions:**

PAH Perioperative management strategies yielded no reductions in postoperative mPAP, but significantly reduced other key clinical outcomes including mean ICU stay and ventilation times in children undergoing cardiac surgery for CHD. Integrating perioperative interventions in the management of PAH may improve overall clinical outcomes, despite minimal impact on mPAP.

**Supplementary Information:**

The online version contains supplementary material available at 10.1186/s13019-026-03893-5.

## Background

Globally, congenital heart disease (CHD) accounts for nearly one-third of major congenital anomalies, with an incidence of 0.8%−1.0% in live-born full-term births and 8.3% in preterm infants [1, 2]. The most prevalent sub-types of CHD in children include ventricular septal defects, atrial septal defects, patent ductus arteriosus, pulmonary stenosis, Tetralogy of Fallot, coarctation, transposition of the great arteries, and aortic stenosis [1]. Approximately 50–60% of children with CHD will require surgical intervention in infancy or early childhood [1]. However, corrective surgery is often not curative, and subsequent surgeries may be required in adulthood due to complications from the primary surgery [1–3]. 

Pulmonary arterial hypertension (PAH), defined by a resting mean pulmonary arterial pressure (mPAP) > 20 mmHg, occurs in around 10% of CHD patients, contributing significantly to morbidity and mortality rates [4–7]. PAH is characterised by systemic-to-pulmonary (left-to-right) shunts and can be identified by right heart catheterisation, which typically arises from an underlying cardiac defect or previous repair [5–8]. Notably, cardiac surgery patients with pre-existing PAH are at an increased risk for developing postoperative complications [9]. Timely surgical repair of CHD is crucial in reducing the likelihood of postoperative PAH and other complications in children [10]. 

In paediatric cardiac surgery, PAH rates serve as a key marker of perioperative morbidity, mortality and long-term survival outcomes [11]. Current perioperative management strategies of PAH include ventilation (to provide adequate oxygenation and avoid acidosis and hypercarbia), optimising haematocrit levels, and adequate analgesia and sedation [12]. Pharmacological interventions such as nitric oxide inhalation, phosphodiesterase (PDE) Type 5 inhibitors (e.g. sildenafil), prostacyclin analogues (e.g. epoprostenol), endothelin receptor antagonists (e.g. bosentan), and inodilators (e.g. milrinone), have shown varying degrees of success [12, 13]. 

Managing PAH, especially in the presence of complex CHD, is challenging due to the risk of exacerbating pre-existing PAH post-operatively or developing persistent chronic PAH [14, 15]. While perioperative management strategies exist, they vary widely across centres [6]. Previous systematic reviews have explored individual pharmacological therapies for preventing or managing post-operative PAH. However, none have comprehensively reviewed the perioperative management of pre-existing PAH due to left-to-right shunting in paediatric CHD patients undergoing cardiac surgery [16, 17]. This systematic review aims to evaluate the effectiveness of perioperative interventions in managing pre-existing PAH and improving clinical outcomes in children with CHD undergoing cardiac surgery.

## Main text

### Methods

#### Study protocol

This review followed the Preferred Reporting Items for Systematic reviews and Meta-Analyses (PRISMA) guidelines [18]. The protocol for this review was registered on.

PROSPERO (CRD42024505271) prior to implementation.

#### Inclusion and exclusion criteria

The PICO framework was used to generate relevant search terms and to develop a comprehensive search strategy (Additional file 1,.docx, Search strategy). Studies were included if the participants were children (under 18 years) with CHD undergoing cardiac surgery, who had been diagnosed with PAH preoperatively. Additionally, eligible studies required implementation of a perioperative intervention to manage PAH compared to a control (no treatment or standard care) and report either PAH or PAP as an outcome. Secondary outcomes included ICU length of stay, ventilation time, pulmonary hypertensive crises, and mortality. Only randomised controlled trials (RCTs) were included, all other study designs including cohort studies, case-control studies, and review articles were excluded. Studies were also limited to human studies available in the English language.

#### Information sources and search strategy

A systematic literature search was conducted using the following databases: Scopus, Medline, Embase, PubMed and Cochrane library. Grey literature was also searched using the National Grey Literature Collection. Additionally, reference lists of identified studies were searched for other relevant papers. The search strategy, which was devised by EI and TR, included the following terms and variants in multiple combinations in each database: “congenital heart disease”, “cardiac surgery” and “pulmonary hypertension” (Additional file 1,.docx, Search strategy).

#### Screening, data extraction and management

The studies identified were managed using EndNote reference management software. Two reviewers (EI and TR) independently selected studies, using the eligibility criteria in a two-stage process: firstly, screening of titles and abstracts, followed by full-text screening. Results were compared, and any disagreements were settled by a third independent reviewer (EAA). Reasoning for excluding trials were fully documented (Additional file 2,.docx, Reasons for excluded studies). Next the reviewers independently conducted data extraction using a standardised data collection form, piloted on three studies and refined for comprehensiveness. Data extraction items included the following: study characteristics, participant characteristics, relevant study outcomes (PAP, ICU stay time, ventilation time, pulmonary hypertensive crises and mortality). If summary data were not published, they were calculated from study data.

#### Quality assessment

Methodological quality of included studies were assessed using the Cochrane RoB-2 assessment tool for RCTs chosen for its validated reliability and robustness assessing risk of bias [19]. This comprehensive tool addressed the following domains: random sequence generation, allocation concealment, blinding of participants and personnel, blinding of outcome assessment, incomplete outcome data, selective outcome reporting and other sources of bias. These domains were classified as “low risk”, “high risk” or “unclear risk” to identify any selection, performance, detection, attrition, reporting and other biases in each study. Two reviewers (EI and TR) independently appraised the methodological quality of included studies using this tool. Publication bias was assessed by generating a funnel plot and by using Egger’s statistical test [20]. 

#### Descriptive and meta-analyses

Outcomes were addressed as pooled mean difference and 95% confidence intervals (CIs). To maintain the random assignment of treatment groups thus providing unbiased comparisons, an intention-to-treat (ITT) analysis was carried out. Meta-analyses were conducted using Stata 18 software to calculate the pooled effect of different interventions used to manage PAH [21]. Pooled mean pulmonary arterial pressure (mPAP) change was calculated for both controls and treatment groups. This data was amalgamated to calculate the pooled mPAP change for both control and treatment groups using a random effects model to assess the effect of perioperative interventions on mPAP. A subgroup analysis was also conducted to compare mPAP change for sildenafil, the most highly reported intervention, compared to other peri-operative interventions. Additionally, the pooled effect of peri-operative interventions on ventilation times and ICU times were calculated. Prevalence rates of pulmonary hypertensive crises and mortality were also calculated for studies that reported these outcomes. Heterogeneity between studies was assessed using the I^2^ statistic (< 25% low, 25–50% moderate and > 50% high heterogeneity) rogeneity) [22]. A meta-analysis was conducted even if high heterogeneity was detected, with results discussed within a heterogeneous context.

## Results

### Search results

**Fig. 1:**
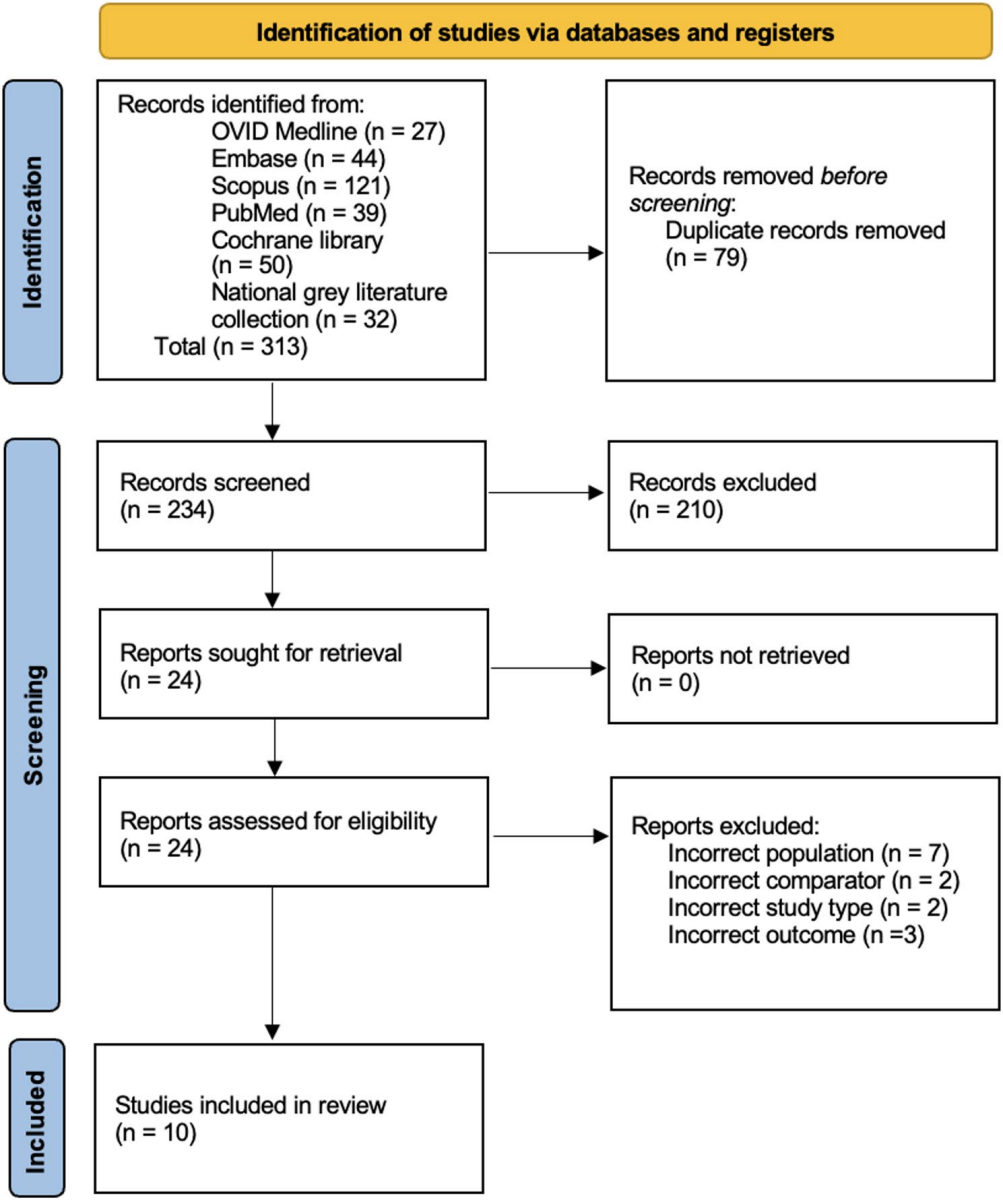
PRISMA flow diagram

313 studies were identified from literature searching, and after applying the eligibility criteria, only 10 studies were included in this review. (Fig. 1**)**. The studies originated from India (*n* = 3) [23, 29, 31], Iran (*n* = 1) [24], China (*n* = 2) [25, 26], Japan (*n* = 2) [27, 32], Turkey (*n* = 1) [28] and the USA (*n* = 1) [30]. Study characteristics are described in Table 1.

**Table 1:** Summary of study characteristics

Study	Country/city	Study setting	Sample size	Age (years) and sex	Intervention	Perioperative PAP difference (mmHg)	Quality assessment
Bhasin et al. 2017 [23]	New Delhi, India	Hospital	60	Control: 1.32 ± 1.09 Treatment: 1.21 ± 1.05 Sex: F = 53.3%	Sildenafil therapy	Control: −18.5 Treatment: −23.1	Low risk of bias across all domains
Bigdelian et al. 2017 [24]	Isfahan, Iran	Hospital	63	Control: 5.4 ± 0.6 Treatment commenced one week preop: 5.4 ± 0.5 Treatment commenced in op: 5.7 ± 0.3. F = 38.1%	Sildenafil therapy	Control: −36.8 Treatment: −44.9	Low risk of bias across all domains
Jiang et al. 2021 [25]	Weihai, China	Hospital	80	Control: 1.63 ± 1.30 Treatment: 1.72 ± 1.12 (Sex not stated)	Treprostinil therapy	Control: −4.04 Treatment: −7.28	Low risk of bias across all domains
Li et al. 2016 [26]	Beijing, China	Hospital	40	Control: 13.8 ± 4.5 Treatment: 13.5 ± 4.2 (Sex not stated)	Novel Goal-Directed Hemodynamic Optimization Therapy	Control: −10 Treatment: −24	High risk of selection, performance and detection bias
Nomura et al. 2013 [27]	Nagoya, Japan	Hospital	13	Control: 0.21 ± 0.19 Treatment: 0.23 ± 0.18 Sex F = 69.2%	Sivelestat	Control: −26.8 Treatment: −23	Low risk of bias across all domains
Onan et al. 2016 [28]	Istanbul, Turkey	Hospital	27	Control: 0.48 ± 0.19 Treatment: 0.65 ± 0.48 F = 51.9%	Intravenous iloprost	Control: −28.4 Treatment: −21	Low risk of bias across all domains
Patel et al. 2020 [29]	Chandigarh, India	Hospital	30	Control: 12 (11.5–24) Treatment: 12 (8.5–15) F = 43.3%	Sildenafil therapy	Control: −22.68 Treatment: −26.96	Low risk of bias across all domains
Russell et al. 1998 [30]	San Francisco, USA	Hospital	36	0.61 (Sex not stated)	Inhaled nitric oxide	Control: 4 Treatment: −8	Low risk of bias across all domains
Sachan et al. 2021 [31]	Ahmedabad, India	Hospital	100	Control: 1.40 ± 1.41 Treatment: 1.13 ± 1.30 F = 59%	Sildenafil therapy	Control: −2.82 Treatment: −3.66	High risk of attrition bias
Umenai et al. 2009 [32]	Kyoto, Japan	Hospital	22	Control: 0.25 Treatment: 0.42 F = 63.6%	Hyperventilation	Control: −29 Treatment: −22	High risk of selection, performance. Detection and attrition bias

M, male; F, female; PAP, pulmonary arterial pressure

Across the included studies, 520 paediatric CHD patients underwent congenital heart surgery. Seven reported the gender of participants, with 52.3% recorded as females, and aged 2.5 months to 12 years [23, 24, 27, 31, 32]. Sildenafil was the most used intervention (*n* = 4) [23, 24, 29, 31], however other interventions included treprostinil [25], iloprost [28], sivelestat [27], nitric oxide [30], and hyperventilation [32]. All studies reported changes in PAP pre- and post-operatively, with additional outcome data based on predefined endpoints such as length of ICU stay, incidence of post-operative pulmonary hypertensive crisis, ventilation time, and mortality (Table 1). Full results of the quality assessment and assessment for publication bias are presented in Additional file 3,.docx, Risk of bias summary and Additional file 4,.docx, Pulmonary arterial pressure respectively.

### Study outcomes- PAH

**Fig. 2:**
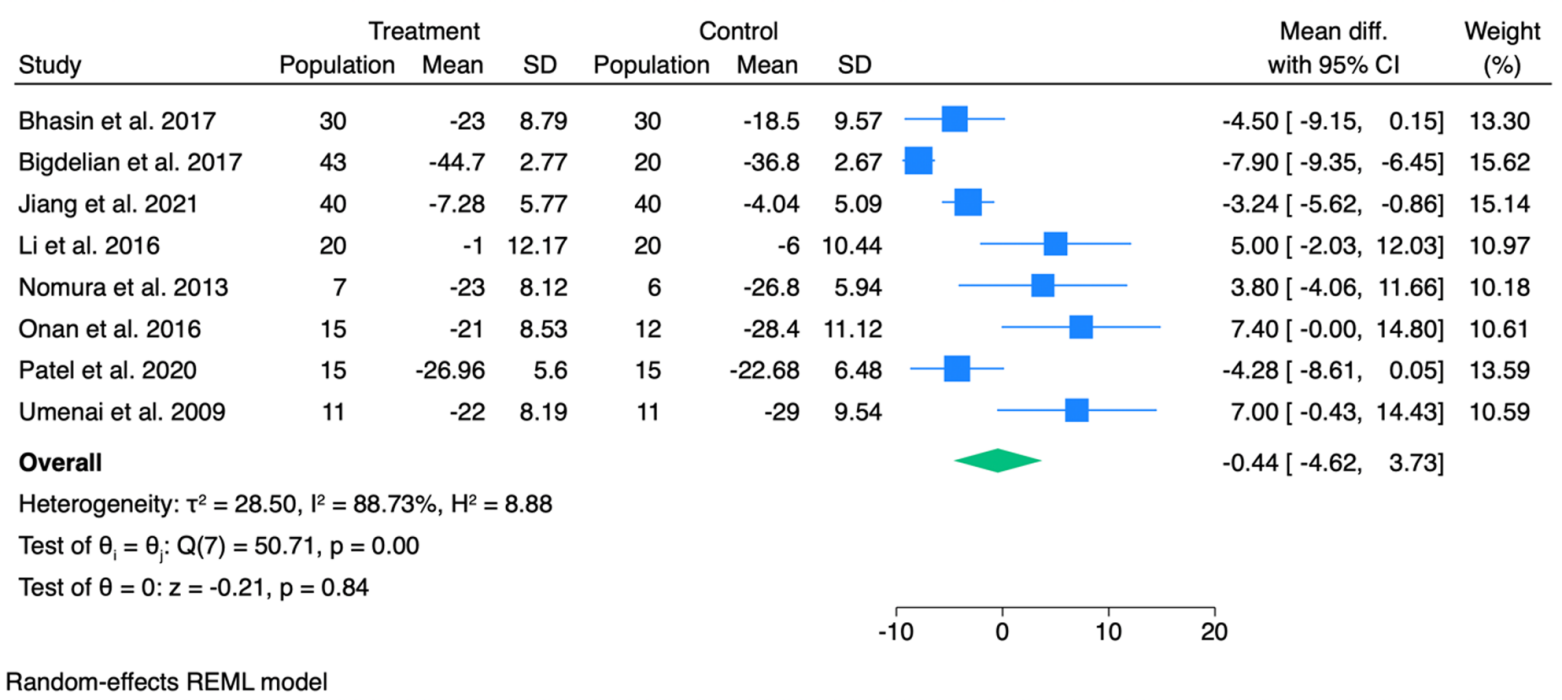
Forest plot of mean difference in pulmonary arterial pressure; CI: confidence interval; SD: standard deviation

**Fig. 3:**
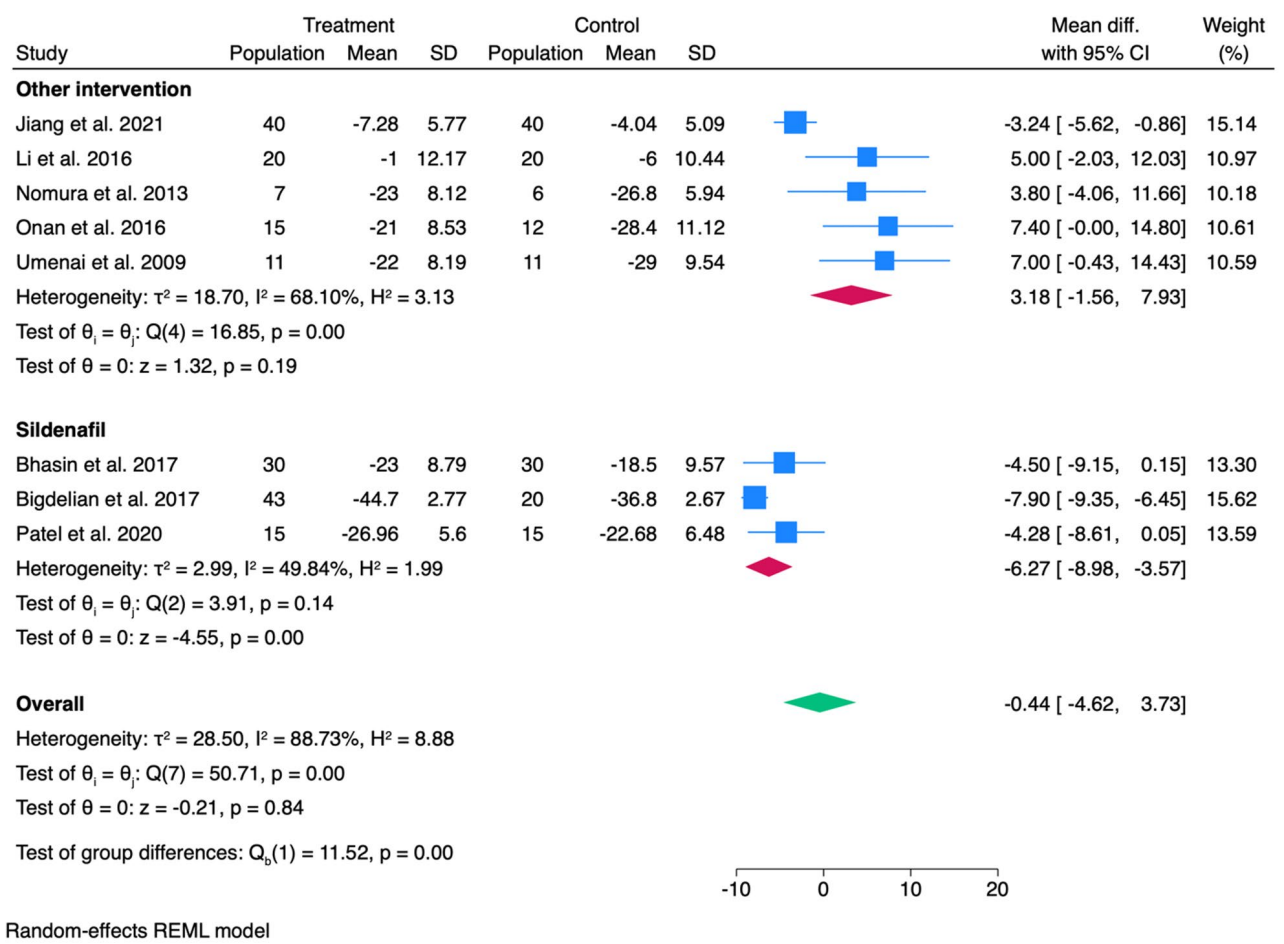
Forest plot of mean difference in pulmonary arterial pressure comparing Sildenafil to other interventions; CI: confidence interval; SD: standard deviation

Overall, the difference in mPAP pre- and post-operatively in the treatment and control groups was statistically insignificant (pooled mean difference, −0.44; 95% CI: −4.62-3.73, *P* = 0.84; I^2^ = 88.73%) (Fig. 2). Compared to other management interventions, sildenafil therapy was found to result in a significant decrease in postoperative mPAP (pooled mean difference, −6.27; 95% CI: −8.982- −3.57, *P* < 0.001; I^2^ = 49.84%) (Fig. 3**)**.

### Study outcomes - other secondary outcomes

**Fig. 4:**
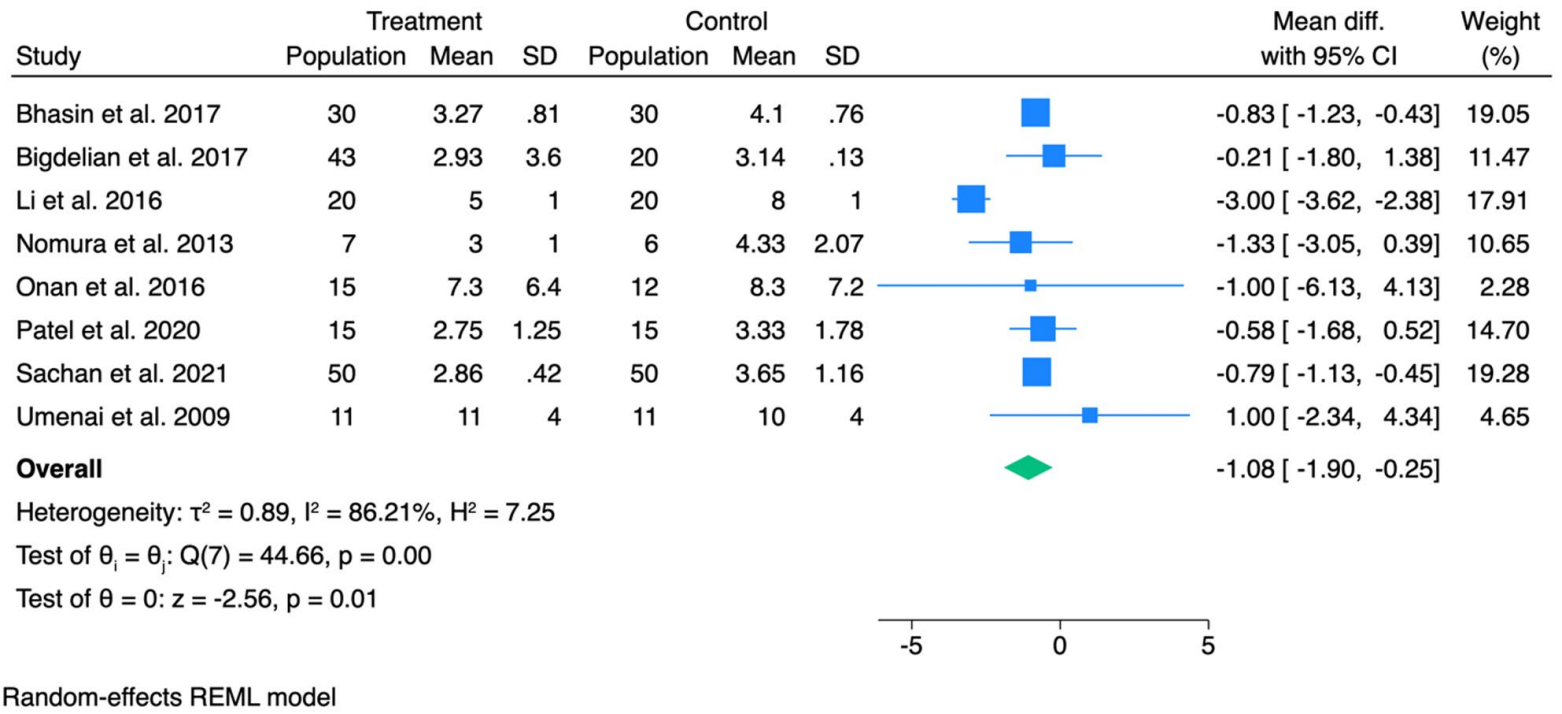
Forest plot of mean difference in ICU stay times; CI: confidence interval; SD: standard deviation

**Fig. 5:**
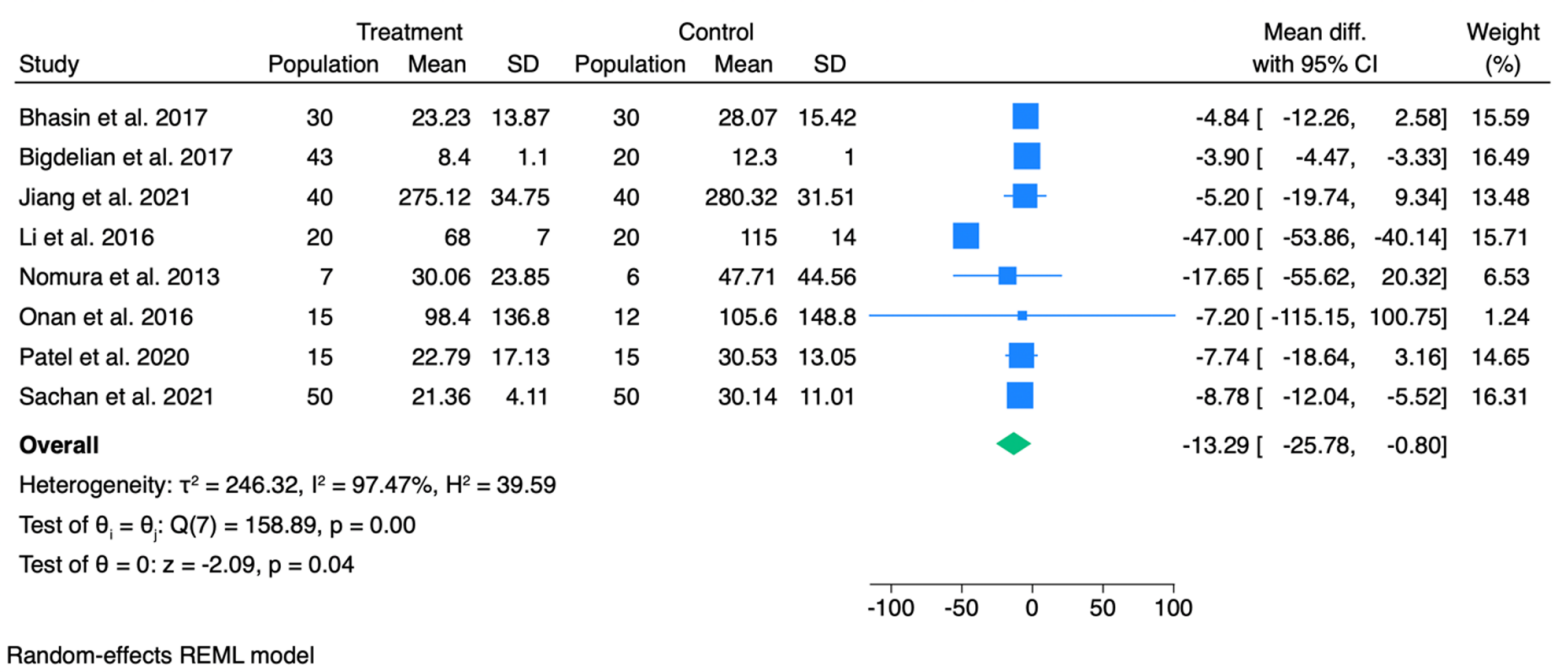
Forest plot of mean difference in ventilation times; CI: confidence interval; SD: standard deviation

The effect of perioperative management strategies on other clinical outcomes were examined. There was a statistically significant difference in reduced ICU times (pooled mean difference, −1.08; 95% CI: −1.90- −0.25, p-value = 0.01; I^2^ = 86.21%) (Fig. 4) and reduced ventilation times (pooled mean difference, −13.29; 95% CI: −25.78 - −0.80, p-value = 0.04; I^2^ = 97.47%) for the treatment groups (Fig. 5).

Notably, from the seven studies that considered pulmonary hypertensive crises as an outcome of interest [23, 24, 27, 29, 31, 32], 11 out of 178 participants (6.18%) and seven out of 204 participants (3.43%) were reported to have hypertensive crises in the control and treatment groups, respectively (Table 2). Additionally, out of the seven studies reporting mortality as an outcome of interest, mortality rates were 1.27% and 0.54% respectively for control and treatment groups (Table 2).

**Table 2:** Pulmonary hypertensive crises *and* mortality

Study	Pulmonary hypertensive crises				Mortality			
	Control events	Control total	Treatment events	Treatment total	Control events	Control total	Treatment events	Treatment total
Bhasin et al. 2017 [23]	1	30	2	30	0	30	0	30
Bigdelian et al. 2017 [24]	3	20	0	43	0	20	0	43
Li et al. 2016 [26]	1	40	0	40	2	20	1	20
Onan et al. 2016 [28]	2	12	4	15	0	12	0	15
Patel et al. 2020 [29]	0	15	0	15	0	15	0	15
Sachan et al. 2021 [31]	3	50	0	50	0	50	0	50
Umenai et al. 2009 [32]	1	11	1	11	0	11	0	11

## Discussion

In this systematic review and meta-analysis eight studies reported data on the effect of peri-operative treatment on mPAP following cardiac surgery for congenital heart disease. Most studies reported a greater decrease in mPAP for participants receiving treatment compared to the control group, however this was not found to be statistically significant. There was high heterogeneity between the included studies potentially due to variance in participant characteristics or due to the differences in the interventions and controls being compared [33]. The meta-analyses also suggest the existence of publication bias due to the asymmetrical distribution of the forest plot and Egger test statistic (*p* < 0.001) (Additional file 2,.docx, Reasons for excluded studies).

The main treatment of choice was sildenafil which was found to reduce mPAP by a greater extent, this difference was found to be statistically significant (*p* < 0.001). Fikri et al. also reported a statistically significant effect with sildenafil use in terms of mPAP and other clinical factors such as ICU length of stay [34]. This suggests that sildenafil could be the treatment of choice in perioperative care in terms of pulmonary hypertension management and optimal clinical outcomes.

Treatment resulted in a reduction of the mean ICU stay and ventilation times compared to the control which was found to be statistically significant. Additionally, of the studies that reported the prevalence of pulmonary hypertensive crises and mortality, both prevalence rates were higher in control groups compared to treatment groups which suggests that the use of perioperative treatment improves the clinical outcomes associated with cardiac surgery in children with CHD and PAH. This impact of perioperative treatment to manage PAH is further highlighted in recent reviews [35, 36]. 

Non-pharmacological strategies also form an essential adjunct to pharmacological therapy in this setting. Optimisation of ventilation parameters is a cornerstone, aiming to maintain adequate oxygenation, prevent hypercarbia, and avoid respiratory and metabolic acidosis – each a potent pulmonary vasoconstrictor (Ivy et al., [11]; Gorenflo et al., [14]). Gentle hyperventilation to lower PaCO₂, combined with high inspired oxygen concentrations can acutely reduce pulmonary vascular resistance, although its use must be balanced against the risks of barotrauma and impaired venous return (Umenai et al., [32]). Careful fluid management is also critical: maintaining optimal preload supports right ventricular (RV) output, while avoiding volume overload that could exacerbate RV dilation and impair left ventricular filling (Abman et al., [5]). Temperature control is another important consideration, as hypothermia can trigger pulmonary vasoconstriction; normothermia should be maintained throughout the perioperative period (Wood et al., 37). Integrated into a structured perioperative protocol, these measures may help stabilise pulmonary haemodynamics, reduce the incidence of crises, and improve short-term outcomes.

Both age at operation and duration of intervention may influence the effectiveness of perioperative strategies in PAH. In this review, studies enrolling predominantly younger children, particularly infants, tended to report larger absolute reductions in PAP, consistent with greater pulmonary vascular reactivity and less fixed vascular remodelling in early life. By contrast, cohorts including older children showed smaller relative changes, potentially reflecting the chronicity of PAH and reduced reversibility of vascular changes. Similarly, the duration of intervention before surgery appeared relevant: studies administering therapy for a week preoperatively, such as Bigdelian et al., [24] achieved some of the largest absolute reductions in mPAP, whereas interventions initiated only intraoperatively or postoperatively tended to yield smaller changes. These patterns suggest that both earlier timing of surgery in the disease course and longer preoperative therapy may enhance pulmonary vasodilation and haemodynamic stability before cardiopulmonary bypass, although dedicated subgroup analyses and prospective studies are needed to confirm these observations.

**Fig. 6:**
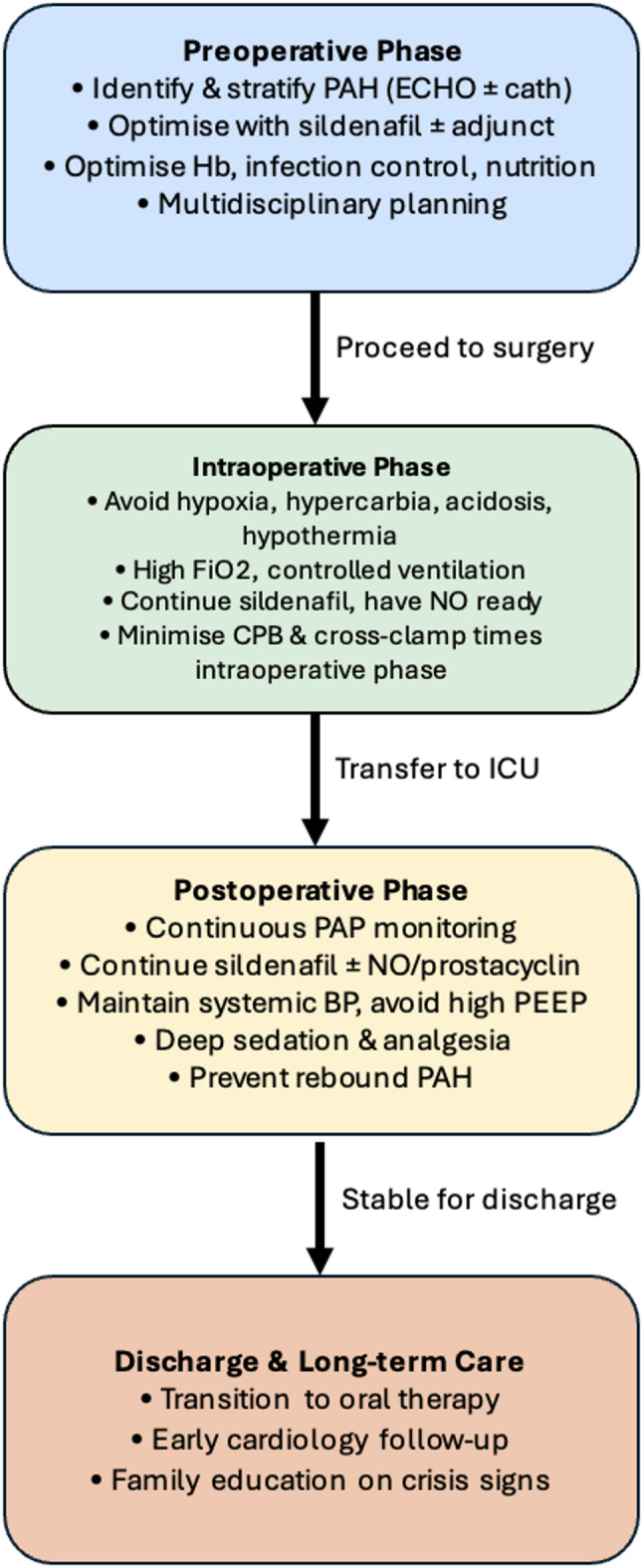
Perioperative management pathway for PAH in children with CHD undergoing cardiac surgery, outlining key interventions from preoperative assessment to discharge; PAH: pulmonary arterial hypertension; ECHO: echocardiography; NO: nitric oxide; CPB: cardiopulmonary bypass; PAP: pulmonary arterial pressure; PEEP: positive end-expiratory pressure

A focused, standardised strategy for managing PAH in children undergoing congenital heart surgery peri-operative management would be effective in changing clinical outcomes. A structured protocol could standardise management across centres (Fig. 6).

Preoperatively, all candidates should be screened for PAH, with catheterisation for severe or uncertain cases, and early optimisation, preferably with sildenafil, alongside correction of anaemia, infection control, and nutritional support. Intraoperatively, teams should aim to minimise pulmonary vascular stress through high FiO₂, lung-protective ventilation, targeted pharmacological support, and reduced CPB and cross-clamp times. Postoperatively, close haemodynamic monitoring, gradual drug weaning, and strict control of sedation, analgesia, and temperature are essential. Discharge planning should include transition to oral therapy, early follow-up, and family education.

Areas for further consideration include whether the route of administration of the intervention influences patient outcomes. Additionally, further randomised controlled trials are needed to compare the effects of different combinations of drugs in managing preoperative PAH. To date, there have not been studies that consider how the type of operation carried out may affect the severity of PAH post-operatively. Other perioperative factors that may influence management such as ventilation and/or the combination of anaesthetics used also must be considered.

This systematic review has several notable strengths. First, a comprehensive search across multiple databases, including grey literature, was conducted to minimise the risk of publication bias and ensure a broad representation of relevant studies. Second, the reference lists of all included articles were manually searched to capture additional eligible studies that might have been overlooked in the initial search. Third, a robust methodology by involving two independent reviewers for screening, data extraction, and quality appraisal, was employed thereby enhancing the reliability and reducing the risk of bias in the review process. Finally, a meta-analysis to quantify the pooled effect was performed, offering a more precise estimate of the outcomes and providing valuable insights for clinical and research contexts.

Limitations of this study include the variation in which PAH and PAP were defined. Two data points (PAP before and after surgery) were used in the meta-analysis, therefore pooled standard deviation rather than standard deviation was calculated. However, two studies included in the systematic review (Russel et al. [30] and Sachin et al. 2021 [31]) reported systolic pulmonary arterial pressure (sPAP) instead of mPAP and therefore could not be included in the meta-analysis. Additionally, the included studies demonstrated high heterogeneity.

To compute the pooled standard deviation, the correlation coefficient value was required, however it was not reported by the included studies. A moderate association correlation coefficient of 0.5 was assumed, a value supported by sensitivity analyses using different correlation coefficient values which revealed no statistically significant differences when compared due to overlapping confidence intervals and inclusion of the null value, 0 (Additional file 4,.docx, Pulmonary arterial pressure) [37]. 

## Conclusion

Although peri-operative management of PAH did not result in a statistically significant reduction in mPAP, these measures did result in a statistically significant reduction of other clinical outcomes such as ICU stay and ventilation times. These findings underscore the importance of optimising peri-operative care, given that prolonged ICU stays, and ventilation can increase costs and the risk of complications, impacting recovery and long-term outcomes. Further research is required to generate standardised management of preoperative PAH in paediatric cardiac surgery cases to improve the surgical outcomes and disease prognosis.

## Supplementary Information

Supplementary Material 1

Supplementary Material 2

Supplementary Material 3

Supplementary Material 4

## Data Availability

The datasets used and/or analysed during the current study are available from the corresponding author on reasonable request.

## Abbreviations

CHD: Congenital heart disease
CI: Confidence interval
ICU: Intensive care unit
mPAP: Mean pulmonary arterial pressure
PAH: Pulmonary arterial hypertension
PAP: Pulmonary artery pressure
RCT: Randomised controlled trial
SD: Standard deviation
sPAP: Systolic pulmonary arterial pressure
