# Supplementary material for: Perioperative management of pulmonary arterial hypertension in children undergoing congenital heart surgery: a systematic review and meta-analysis
Source: J Cardiothorac Surg. 2026 Apr 25;21:420. doi: 10.1186/s13019-026-03893-5 (PMC13267292; doi:10.1186/s13019-026-03893-5)
Supplement: Supplementary file 1 — Supplementary Material 1 [file 13019_2026_3893_MOESM1_ESM.docx]

**Additional file 1 - Search strategy**

*1.1 Search terms*

Table 1: Search Terms

| **Area** | **Search Terms** |
| --- | --- |
| Paediatric | Paediatric OR pediatric OR Infant OR neonate OR newborn OR new-born OR baby OR babies OR toddler OR child OR children OR childhood OR minor OR kid OR teenager OR teen OR juvenile OR adolescent OR youth OR young OR preterm OR premature OR offspring |
| Congenital heart disease | Congenital heart disease OR heart defect OR heart abnormality OR heart malformation OR ventricular septal defect OR atrial septal defect OR pulmonary stenosis OR patent ductus arteriosus OR tetralogy of fallot OR coarctation OR transposition of the great arteries OR aortic stenosis |
| Cardiac surgery | Cardiac surgery OR heart surgery OR cardiothoracic surgery OR thoracic surgery OR cardiac surgical procedure OR thoracic surgical procedure |
| Pulmonary hypertension | Pulmonary hypertension AND pulmonary arterial hypertension OR Eisenmenger syndrome |
| Randomised controlled trial | Randomised controlled trial OR crossover procedure OR blind procedure OR randomised clinical trial OR randomized clinical trial OR controlled clinical trial |

*1.2. Search strategy*

Example: Ovid MEDLINE(R) ALL <1946 to November 14, 2023>

1 Pediatrics/ or Paediatric.mp. 129209

2 pediatric.mp. or Pediatrics/ 424889

3 Infant/ 871827

4 neonate.mp. or Infant, Newborn/ 688112

5 newborn.mp. or Infant, Newborn/ 829523

6 new-born.mp. 4472

7 baby.mp. 46305

8 babies.mp. 42654

9 toddler.mp. 5926

10 child.mp. 2394925

11 children.mp. or Child/ 2375634

12 childhood.mp. 308537

13 minor.mp. or Minors/ 264800

14 kid.mp. 2624

15 teenager.mp. or Adolescent/ 2227892

16 teen.mp. or Adolescent/ 2228318

17 juvenile.mp. 97703

18 adolescent.mp. 2269257

19 youth.mp. or Adolescent/ 2256002

20 young.mp. 1501275

21 preterm.mp. 95191

22 premature.mp. 226922

23 offspring.mp. 88403

24 1 or 2 or 3 or 4 or 5 or 6 or 7 or 8 or 9 or 10 or 11 or 12 or 13 or 14 or 15 or 16 or 17 or 18 or 19 or 20 or 21 or 22 or 23 5800309

25 Congenital heart disease.mp. or Heart Defects, Congenital/ 76741

26 heart defect.mp. 3907

27 heart abnormality.mp. or Heart Defects, Congenital/ 60169

28 heart malformation.mp. 435

29 ventricular septal defect.mp. or Heart Septal Defects, Ventricular/ 19402

30 atrial septal defect.mp. or Heart Septal Defects, Atrial/ 17781

31 pulmonary stenosis.mp. or Pulmonary Valve Stenosis/ 8485

32 patent ductus arteriosus.mp. or Ductus Arteriosus, Patent/ 13757

33 tetralogy of Fallot.mp. or "Tetralogy of Fallot"/ 13595

34 Aortic Coarctation/ or coarctation.mp. 13287

35 "Transposition of Great Vessels"/ or transposition of the great arteries.mp. 9525

36 aortic stenosis.mp. or Aortic Valve Stenosis/ 40370

37 25 or 26 or 27 or 28 or 29 or 30 or 31 or 32 or 33 or 34 or 35 or 36 174951

38 Cardiac surgery.mp. or Thoracic Surgery/ 63701

39 heart surgery.mp. or Thoracic Surgery/ 30373

40 cardiothoracic surgery.mp. 3877

41 cardiac surgical procedure.mp. or Cardiac Surgical Procedures/ 61154

42 38 or 39 or 40 or 41 108980

43 pulmonary hypertension/ 39340

44 pulmonary arterial hypertension.mp. 16134

45 Eisenmenger syndrome.mp. or Eisenmenger complex/ 1529

46 43 or 44 or 45 47087

47 randomised controlled trial.mp. or randomized controlled trial/ 615555

48 crossover procedure.mp. or crossover procedure/ 49

49 blind procedure.mp. 369

50 randomised clinical trial.mp. 4448

51 randomized clinical trial.mp. 41095

52 controlled clinical trial/ 95456

53 47 or 48 or 49 or 50 or 51 or 52 721182

1. 24 and 37 and 42 and 46 and 53 27
