# Supplementary material for: Perioperative management of pulmonary arterial hypertension in children undergoing congenital heart surgery: a systematic review and meta-analysis
Source: J Cardiothorac Surg. 2026 Apr 25;21:420. doi: 10.1186/s13019-026-03893-5 (PMC13267292; doi:10.1186/s13019-026-03893-5)
Supplement: Supplementary file 2 — Supplementary Material 2 [file 13019_2026_3893_MOESM2_ESM.docx]

**Additional file 3 - Risk of Bias Summary**


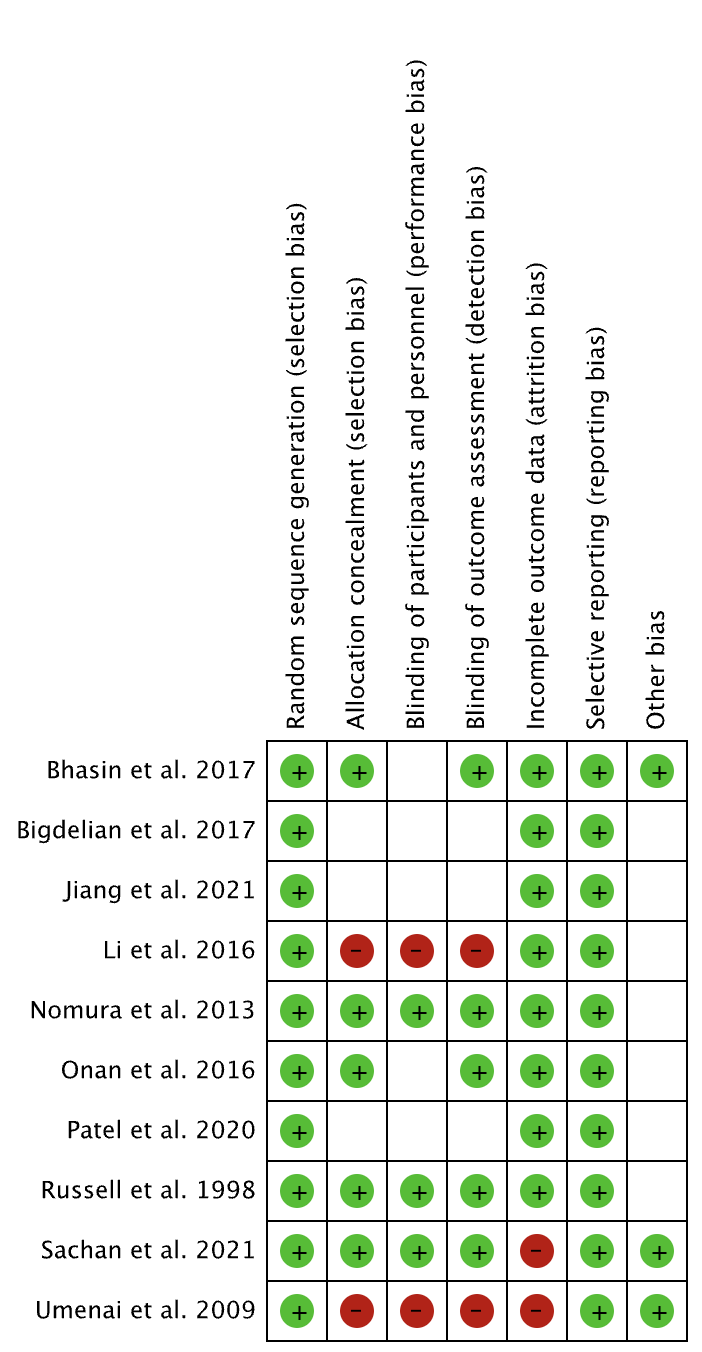


*Figure 1. risk of bias*

“ + ” means low risk of bias; “-“ means high risk of bias
