# Supplementary material for: Perioperative management of pulmonary arterial hypertension in children undergoing congenital heart surgery: a systematic review and meta-analysis
Source: J Cardiothorac Surg. 2026 Apr 25;21:420. doi: 10.1186/s13019-026-03893-5 (PMC13267292; doi:10.1186/s13019-026-03893-5)
Supplement: Supplementary file 3 — Supplementary Material 3 [file 13019_2026_3893_MOESM3_ESM.docx]

**Additional file 2 - Reasons for excluded studies**

Table 2: Reasons for exclusions

| **Study** | **Reason for exclusion** |
| --- | --- |
| Bando K, Turrentine MW, Vijay P, Sharp TG, Sekine Y, Lalone BJ, et al. Effect of modified ultrafiltration in high-risk patients undergoing operations for congenital heart disease. Ann Thorac Surg. 1998;66(3):821-7; discussion 8. | Incorrect outcome |
| Barnwal NK, Umbarkar SR, Sarkar M, Dias R. Randomized comparative study of intravenous infusion of three different fixed doses of milrinone in pediatric patients with pulmonary hypertension undergoing open heart surgery. Annals of Cardiac Anaesthesia. 2017;20(3):318-22. | Incorrect comparator |
| Beghetti M, Channick RN, Chin KM, Di Scala L, Gaine S, Ghofrani HA, et al. Selexipag treatment for pulmonary arterial hypertension associated with congenital heart disease after defect correction: insights from the randomised controlled GRIPHON study. Eur J Heart Fail. 2019;21(3):352-9. | Incorrect study type |
| Day RW, Hawkins JA, McGough EC, Crezeé KL, Orsmond GS. Randomized controlled study of inhaled nitric oxide after operation for congenital heart disease. Ann Thorac Surg. 2000;69(6):1907-12; discussion 13. | Incorrect population |
| El Midany AA, Mostafa EA, Azab S, Hassan GA. Perioperative sildenafil therapy for pulmonary hypertension in infants undergoing congenital cardiac defect closure. Interact Cardiovasc Thorac Surg. 2013;17(6):963-8. | Incorrect comparator |
| Miller O, Tang SF, Keech A, Pigott NB, Beller E, Celermajer DS. Inhaled nitric oxide and prevention of pulmonary hypertension after congenital heart surgery: A randomised double-blind study. Lancet. 2000;356(9240):1464-9. | Incorrect outcome |
| Peiravian F, Amirghofran AA, Borzouee M, Ajami GH, Sabri MR, Kolaee S. Oral sildenafil to control pulmonary hypertension after congenital heart surgery. Asian Cardiovascular and Thoracic Annals. 2007;15(2):113-7. | Incorrect population |
| Sharma VK, Joshi S, Joshi A, Kumar G, Arora H, Garg A. Does intravenous sildenafil clinically ameliorate pulmonary hypertension during perioperative management of congenital heart diseases in children?-A prospective randomized study. Annals of Cardiac Anaesthesia. 2015;18(4):510-6. | Incorrect outcome |
| Silvera Ruiz S, Grosso CL, Tablada M, Cabrera M, Dodelson de Kremer R, Juaneda E, et al. Efficacy of citrulline supplementation to decrease the risk of pulmonary hypertension after congenital heart disease surgery. A local experience. Revista de la Facultad de Ciencias Medicas (Cordoba, Argentina). 2020;77(4):249-53. | Incorrect population |
| Smith HAB, Canter JA, Christian KG, Drinkwater DC, Scholl FG, Christman BW, et al. Nitric oxide precursors and congenital heart surgery: A randomized controlled trial of oral citrulline. Journal of Thoracic and Cardiovascular Surgery. 2006;132(1):58-65. | Incorrect population |
| Takahashi K, Mori Y, Yamamura H, Nakanishi T, Nakazawa M. Effect of beraprost sodium on pulmonary vascular resistance in candidates for a Fontan procedure: a preliminary study. Pediatr Int. 2003;45(6):671-5. | Incorrect study type |
| Vassalos A, Peng E, Young D, Walker S, Pollock J, Macarthur K, et al. Pre-operative sildenafil and pulmonary endothelial-related complications following cardiopulmonary bypass: a randomised trial in children undergoing cardiac surgery. Anaesthesia. 2011;66(6):472‐80. | Incorrect population |
| Xu Z, Zhu L, Liu X, Gong X, Gattrell W, Liu J. Iloprost for children with pulmonary hypertension after surgery to correct congenital heart disease. Pediatric pulmonology. 2015;50(6):588-95. | Incorrect population |
| Zhang CY, Ma ZS, Ma LL, Wang LX. Effect of prostaglandin E1 inhalation on pulmonary hypertension following corrective surgery for congenital heart disease. Experimental and clinical cardiology. 2013;18(1):13‐6. | Incorrect population |
