# Supplementary material for: Perioperative management of pulmonary arterial hypertension in children undergoing congenital heart surgery: a systematic review and meta-analysis
Source: J Cardiothorac Surg. 2026 Apr 25;21:420. doi: 10.1186/s13019-026-03893-5 (PMC13267292; doi:10.1186/s13019-026-03893-5)
Supplement: Supplementary file 4 — Supplementary Material 4 [file 13019_2026_3893_MOESM4_ESM.docx]

**Additional file 4 – Pulmonary arterial pressure**

4*.1. Funnel plot of mean difference in pulmonary arterial pressure*
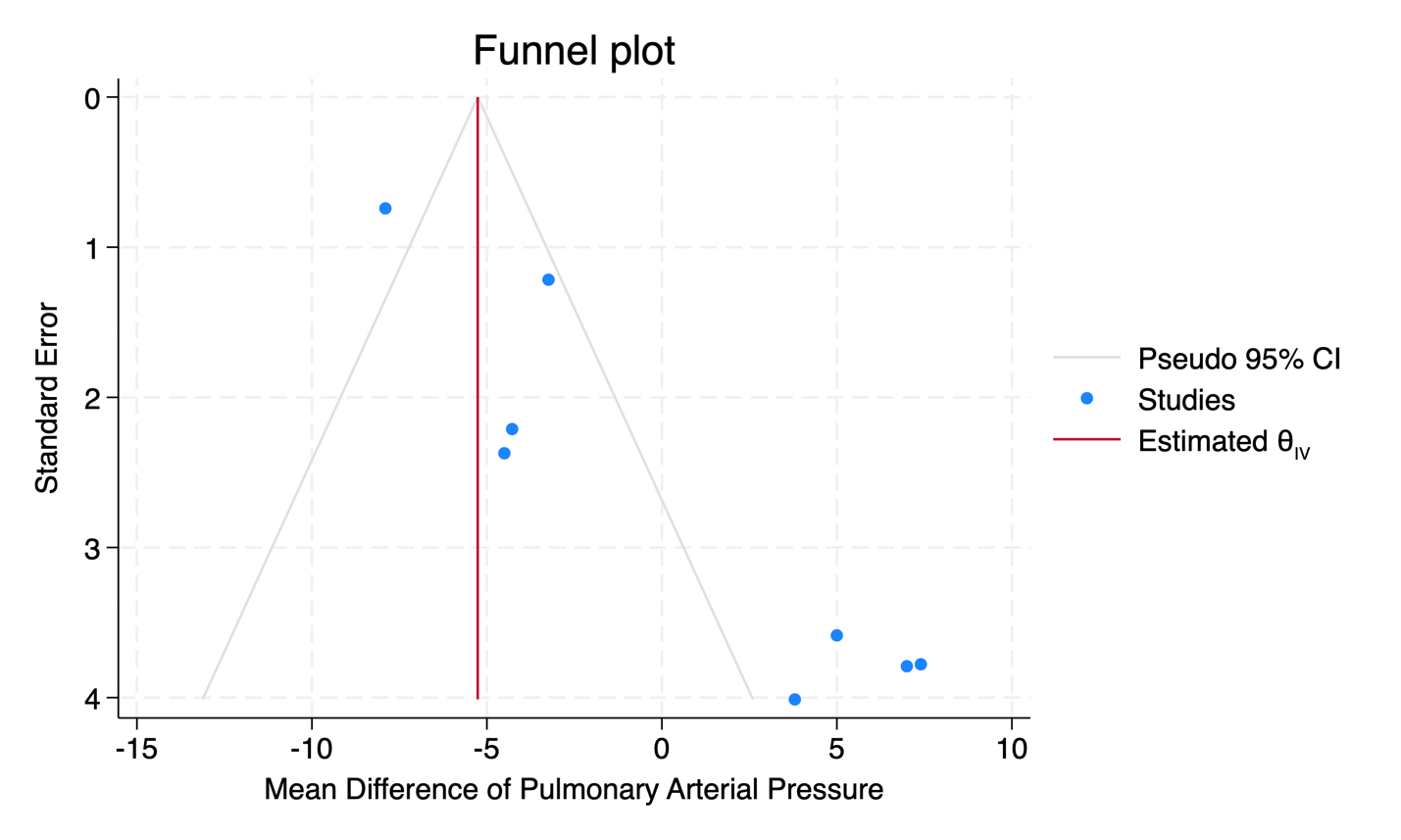


*Figure 2. Funnel plot of mean difference in pulmonary arterial pressure*

The Egger's test statistic was 4.68 (p<0.001), indicating potential publication bias.

- 1. *Sensitivity analysis of mean difference in pulmonary arterial pressure*


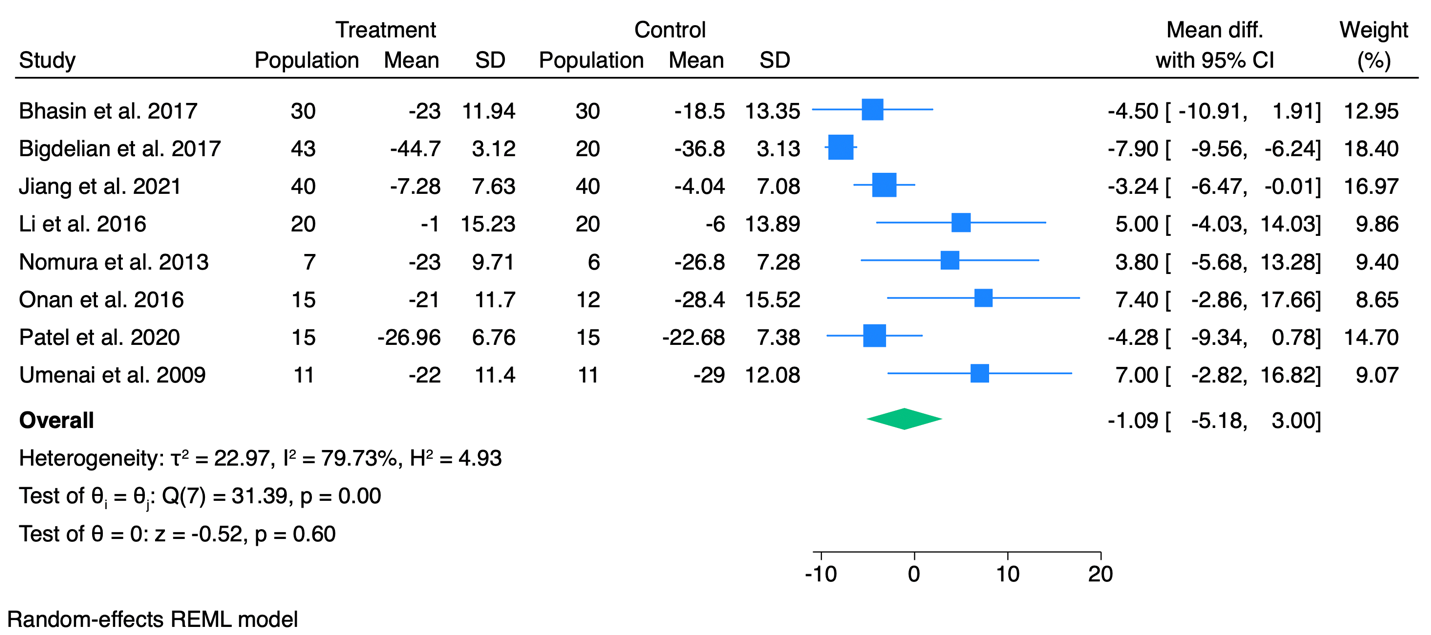


*Figure 3. Forest plot of mean difference in pulmonary arterial pressure with correlation coefficient =0*


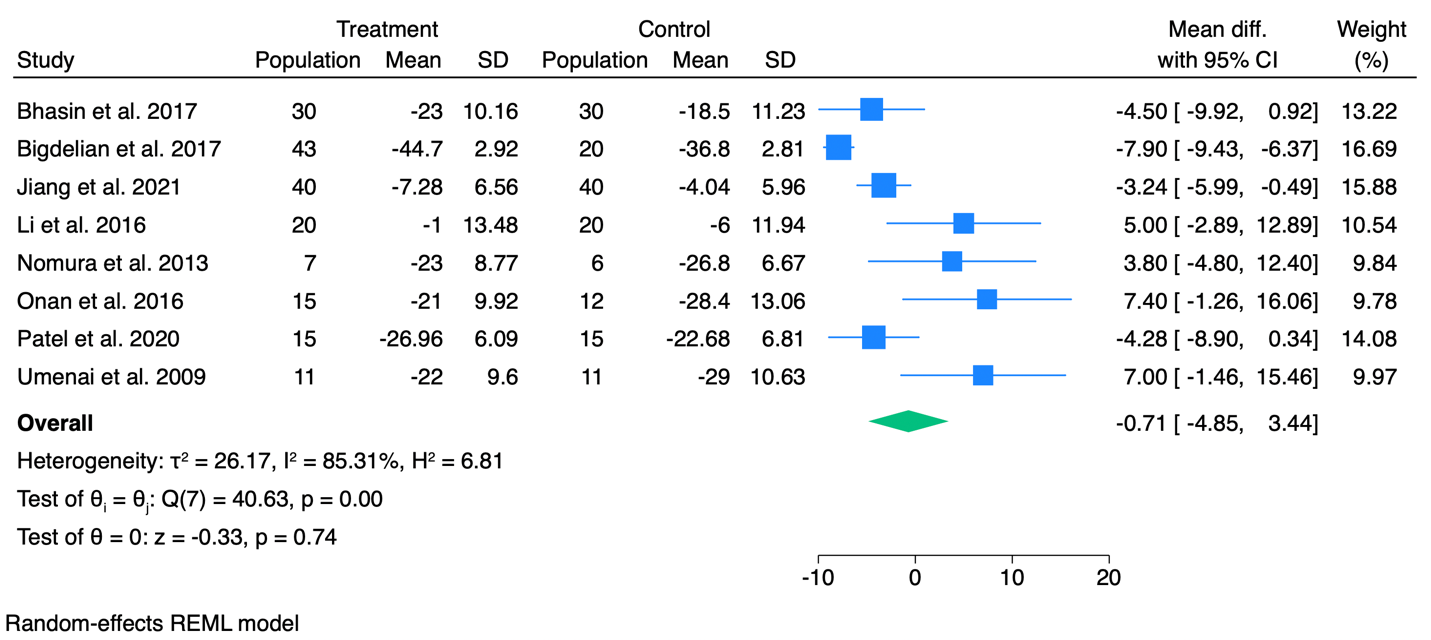


*Figure 4. Forest plot of mean difference in pulmonary arterial pressure with correlation coefficient =0.3*


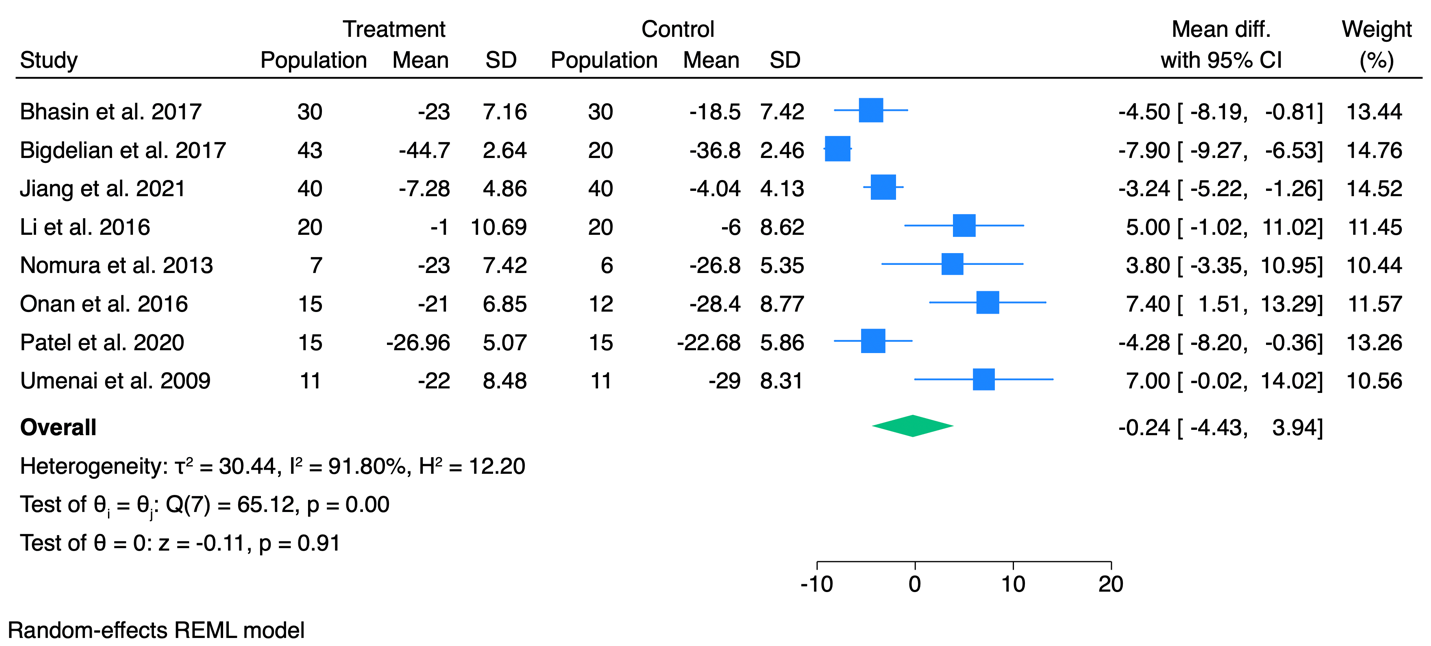


*Figure 5. Forest plot of mean difference in pulmonary arterial pressure with correlation coefficient =0.7*
